# Role of Sigma Receptors in Methamphetamine-Induced Neurotoxicity

**DOI:** 10.2174/157015911795016930

**Published:** 2011-03

**Authors:** Nidhi Kaushal, Rae R Matsumoto

**Affiliations:** Department of Basic Pharmaceutical Sciences, School of Pharmacy, West Virginia University, Morgantown, WV 26506, USA

**Keywords:** Methamphetamine, sigma receptors, oxidative stress, endoplasmic reticulum, mitochondria, neurotoxicity, dopamine, glutamate.

## Abstract

Methamphetamine (METH) is a widely abused substance world over. Currently, there is no effective pharmacotherapy to treat its effects. This necessitates identification of potential novel therapeutic targets. METH interacts with sigma (σ) receptors at physiologically relevant micromolar concentrations. In addition, σ receptors are present in organs like the brain, heart, and lungs at which METH acts. Additionally, σ receptors have been implicated in various acute and subchronic effects like locomotor stimulation, development of sensitization and neurotoxicity, where σ receptor antagonists attenuate these effects. σ Receptors may also have a role in METH-induced psychiatric complications such as depression, psychosis, cognitive and motor deficits. The neurotoxic effects of METH, which are cause for concern, can be prevented by σ receptor antagonists in mice. Mechanistically, METH-induced neurotoxicity involves factors like dopamine release, oxidative stress, endoplasmic reticulum stress, activation of mitochondrial death cascades, glutamate  release, apoptosis, microglial activation, and hyperthermia. This review compiles studies from the literature that suggests an important role for σ receptors in many of the mechanisms of METH-induced neurotoxicity.

## METHAMPHETAMINE (METH): A DRUG OF ABUSE

Methamphetamine (METH) is a widely abused substance due to its ease of synthesis from over the counter drugs and its long duration of action [1]. Due to these factors, it ranks second worldwide after Cannabis as the most extensively abused drug, with an estimated 15-16 million users [2]. Apart from its immediate stimulant effects such as euphoria and enhanced energy, METH causes harmful effects upon exposure to repeated or large doses. Some of these harmful effects include hyperthermia, addiction, altered neurological and cognitive functions, psychosis, depression, and neurodegeneration [2]. 

The neurodegenerative effects are causes of concern as neuroimaging and postmortem studies in humans depict damage to dopaminergic and serotonergic systems, which can be measured as depletions in dopamine transporters (DAT), serotonin transporters (SERT), and dopamine and serotonin levels [2]. There is evidence that these neurodegenerative effects are implicated in some of the psychiatric symptoms observed due to METH [3]. However, the mechanisms underlying these neurodegenerative effects are still not clearly understood. Also, no effective treatment exists for these effects of METH. 

## METH AND SIGMA RECEPTORS

Exploration of a novel target to develop medications against, and to better understand the mechanisms of METH-induced neurotoxicity led to the discovery that METH interacts with sigma (σ) receptors at physiologically relevant concentrations [4]. σ Receptors are a unique group of drug binding sites [5]. They are found extensively in the brain and periphery [5], with two established subtypes: σ-1 and σ-2. METH binds to σ-1 receptors with a K_i_ of 2 μM and σ-2 receptors with a K_i_ of 47 μM [4]. σ-1 Receptors have been cloned and are not homologous to any known protein except for sharing 30% homology with fungal sterol isomerase [5]. Following the binding of ligands, σ-1 receptors have been shown to operate *via *protein-protein interactions and translocation between subcellular compartments through chaperone-like functions [5-7]. In contrast, σ-2 receptors have not yet been cloned. They are localized in lipid rafts, plasma membrane, endoplasmic reticulum (ER), mitochondria, and lysosomes of cells [8, 9]. σ-2 Receptors are highly upregulated in tumor cell lines and have a role in cell death signaling *via *sphingolipid products [10]. 

σ Receptors seem to play an important role in many of the effects of METH. They are present in the organs that mediate the actions of METH (e.g. brain, heart, lungs) [5]. In the brain, METH acts primarily on the dopaminergic system to cause acute locomotor stimulant, subchronic sensitized, and neurotoxic effects. σ Receptors are present on dopaminergic neurons and their activation stimulates dopamine synthesis and release [11-13]. σ-2 Receptors modulate DAT and the release of dopamine *via *protein kinase C (PKC) and Ca^2+^-calmodulin systems [14]. 

σ-1 Receptor antisense and antagonists have been shown to block the acute locomotor stimulant effects of METH [4]. Repeated administration or self administration of METH has been shown to upregulate σ-1 receptor protein and mRNA in various brain regions including the substantia nigra, frontal cortex, cerebellum, midbrain, and hippocampus [15, 16]. Additionally, σ receptor antagonists like MS377 (R)-(1)-1-(4-chlorophenyl)-3-[4-(2-methoxyethyl)piperazin-1-yl]methyl-2-pyrrolidinone-L-tartrate) and BMY14802 (alpha-(4-fluorophenyl)-4-(5-fluoro-2-pyrimidinyl)-1-piperazine-butanol) prevent the development of behavioral sensitization to METH [17, 18]. 

Clinically, METH users display cognitive deficits, depression, anxiety, psychotic symptoms, and motor deficits similar to Parkinson’s disease [2]. Apart from being present in brain regions associated with these disorders [19, 20], σ receptors have also been implicated in cognitive dysfunctions, depression, anxiety, schizophrenia, and Parkinson’s disease [21, 22]. Some of the observed psychiatric symptoms especially cognitive dysfunction seems to arise from the neurodegenerative effects of METH [3].

METH causes neurodegenerative/neurotoxic effects following high or repeated dosing, which has been manifested as dopaminergic and serotonergic nerve terminal degeneration (measured as depletions in neurotransmitter levels and decreases in monoamine transporter levels) as well as more recently, apoptotic cell death [23]. These effects have been observed in humans as well as in animal models involving mice, rats, and monkeys [2]. σ Receptor antagonists like AC927 (N-phenethylpiperidine oxalate) prevent the neurotoxic effects of METH in Swiss Webster mice, including METH-induced depletions in dopamine and DAT levels in striatal brain regions [24].

Mechanistically, the neurotoxic effects of METH seem to involve the activation of certain events such as dopamine release, oxidative stress, ER stress and mitochondrial death cascades, excitotoxicity, microglial activation, and hyperthermia [2]. In addition, compelling evidence in the literature indicate that apart from being a direct target of METH, σ receptors play an important role in the modulation of other neurotransmitter systems and downstream events involved in METH-induced neurotoxicity. Systematic mechanistic studies, however, have not been conducted in the context of METH. The rest of the review is focused on the various downstream events where σ receptors may potentially modulate the effects of METH. In those instances where the role of an individual subtype of σ receptor is known, the subtype is specified. Otherwise, the term σ receptor, with no subtype designation, is used.

### Dopamine

METH acts through DAT and vesicular monoamine transporter 2 (VMAT2) to cause excessive release of dopamine into the cytoplasm and synapse [25, 26], which is thought to be one of the initiating factors in METH’s neurotoxic cascade. σ Receptor agonists have been shown to facilitate dopamine release, through both σ-1 and σ-2 receptors [11-14].

### Reactive Oxygen Species

After this excessive dopamine release, auto-oxidation reactions lead to the formation of harmful reactive oxygen species (ROS) including dopamine quinones, superoxide radicals, hydroxyl radicals, and hydrogen peroxide [2]. These ROS can damage neurons and surrounding cells *via *oxidative damage to cellular components such as lipids, proteins, and DNA [2]. 

σ-2 Receptor activation has been shown to cause the release of ROS in tumor cells [27]. In contrast, σ receptor antagonism affords protection in brain ischemia, a condition in which ROS are generated [28]. These studies indicate a potential connection between σ receptors and oxidative stress mechanisms of METH-induced neurotoxicity. 

### ER Stress, Calcium and Mitochondrial Death Cascades

METH causes ER stress and activation of mitochondrial death cascades partly due to increases in oxidative stress in the cell [29]. ER stress causes disturbances in cellular calcium homeostasis [30]. ER stress and calcium dysregulation contributes to cell death observed due to METH [29]. Sustained ER stress and excessive calcium efflux in turn can contribute to METH-induced apoptosis *via *mitochondrial pore permeabilization [29, 31]. 

σ Receptors are located on the ER and mitochondria [8, 22]. σ-1 Receptors have been shown to function as chaperones at the mitochondrial-associated ER membrane (MAM). At MAM, σ-1 receptors play an important role in regulating calcium levels *via *inositol triphosphate (IP_3_) receptors and maintaining homeostasis in the cell against oxidative stress and perturbations in calcium [7]. On the other hand, σ-2 receptor activation has been shown to cause a biphasic flux of calcium from the ER and mitochondrial stores which leads to apoptotic cell death [32, 33].

### Apoptosis

METH causes apoptotic cell death in certain brain areas including the striatum, cortex, and hippocampus [34]. The apoptotic process can be either caspase-dependent or independent [29].

σ-2 Receptor activation has been shown to cause apoptosis in tumor cells *via *caspase-dependent and independent processes, depending on the cell type [10, 35]. σ-2 Receptor activation has also been shown to cause apoptotic cell death in tumor cells *via *alterations in cellular sphingolipid levels [36]. In contrast, σ-1 receptor agonists have been shown to have neuroprotective effects by regulating intracellular calcium levels and preventing activation of pro-apoptotic genes in retinal ganglion cells [37]. σ-1 Receptor agonists have also been reported to preserve protective genes like bcl-2 in a cerebral focal ischemia model [38].

### Glutamate and Excitotoxicity

Glutamate release and excitotoxic mechanisms have also been implicated in METH-induced neurotoxicity [2]. σ Receptors have an important role in modulating the glutamatergic N–methyl-D-aspartate (NMDA) system and display neuroprotective effects against excitotoxic mechanisms in ischemic stroke models, midbrain and cortical dopaminergic neurons, and retinal ganglion cells [39-42].

### Microglial Activation

Recent studies have underscored the role of accessory cells like microglia in METH-induced neurotoxicity [43, 44]. Microglial activation has been observed in areas of the brain that have been damaged by METH [43, 44]. Drugs that attenuate neurotoxic effects also suppress microglial activation [2]. However, the mechanisms by which microglia influence METH’s neurotoxic effects are still not very clear. Release of cytokines and ROS have been suggested as possible contributing mechanisms [2]. σ Receptors are found on microglia and the σ receptor agonist di-o-tolylguanidine has been shown to suppress microglial activation [45]; the subtype most likely involved in this effect is σ-1, although experiments to specifically address this issue have yet to be performed. 

### Hyperthermia

METH increases body temperature, which is known to exacerbate neurotoxic effects [46]. σ Receptors are present in the hypothalamus and can modulate body temperature [19, 47]. σ Receptor antagonists have been shown to decrease METH-induced hyperthermia, which is consistent with an overall important and beneficial role for mitigating detrimental effects of METH. However, it is important to note that decreasing body temperature is not the only mechanism by which σ receptors afford neuroprotective effects. This is because, firstly, σ receptors have already been shown to have an important role in cell death processes/mechanisms, some of which are also involved in METH-induced neurotoxicity. Secondly, σ receptor antagonists have been shown to attenuate neurotoxic effects in a cell culture system where the temperature was kept constant [24]. Thirdly, although temperature seems to play an important role in the neurotoxic effects of METH, it is not the only mechanism of METH’s toxicity. Elevated ambient temperature by itself does not show toxicity [46]; pharmacological manipulations like nNOS inhibitor attenuate toxicity but do not decrease temperature [48]; and finally, reserpine causes hypothermia and reduces METH-induced hyperthermia but does not attenuate neurotoxicity associated with METH [49].

In summary, the available information indicates that σ-2 receptor agonism and σ-1 receptor antagonism mediate cytotoxicity. Therefore, at neurotoxic doses, METH most likely produces its damage through σ-2 receptor agonism. At low stimulant doses, METH appears to act as an agonist at σ-1 receptors, with pharmacological antagonists and antisense oligonucleotides attenuating METH-induced locomotor activity [4]. Although σ-1 agonism would indicate the potential for neuroprotective effects, at higher concentrations, METH does not produce functional effects like a typical σ-1 receptor agonist as demonstrated by a recent study of its mechanism as a chaperone [7]. When considered together, the data suggest that with regard to the actions of METH, cytotoxic effects through σ-2 receptors dominates over any protective actions that may be afforded *via *the σ-1 subtype. Although, additional studies to further delineate specific mechanisms are still needed, the information in the literature suggest σ receptors, particularly the σ-2 subtype, as a very promising target for developing medications against and understanding the mechanism of METH-induced neurotoxicity along with the other adverse effects. 
